# Identification of Candidate Agents Active against *N. ceranae* Infection in Honey Bees: Establishment of a Medium Throughput Screening Assay Based on *N. ceranae* Infected Cultured Cells

**DOI:** 10.1371/journal.pone.0117200

**Published:** 2015-02-06

**Authors:** Sebastian Gisder, Elke Genersch

**Affiliations:** Institute for Bee Research, Department of Molecular Microbiology and Bee Diseases, Hohen Neuendorf, Germany; Goethe University Frankfurt, GERMANY

## Abstract

Many flowering plants in both natural ecosytems and agriculture are dependent on insect pollination for fruit set and seed production. Managed honey bees (*Apis mellifera*) and wild bees are key pollinators providing this indispensable eco- and agrosystem service. Like all other organisms, bees are attacked by numerous pathogens and parasites. *Nosema apis* is a honey bee pathogenic microsporidium which is widely distributed in honey bee populations without causing much harm. Its congener *Nosema ceranae* was originally described as pathogen of the Eastern honey bee (*Apis cerana*) but jumped host from *A. cerana* to *A. mellifera* about 20 years ago and spilled over from *A. mellifera* to *Bombus* spp. quite recently. *N. ceranae* is now considered a deadly emerging parasite of both Western honey bees and bumblebees. Hence, novel and sustainable treatment strategies against *N. ceranae* are urgently needed to protect honey and wild bees. We here present the development of an *in vitro* medium throughput screening assay for the identification of candidate agents active against *N. ceranae* infections. This novel assay is based on our recently developed cell culture model for *N. ceranae* and coupled with an RT-PCR-ELISA protocol for quantification of *N. ceranae* in infected cells. The assay has been adapted to the 96-well microplate format to allow automated analysis. Several substances with known (fumagillin) or presumed (surfactin) or no (paromomycin) activity against *N. ceranae* were tested as well as substances for which no data concerning *N. ceranae* inhibition existed. While fumagillin and two nitroimidazoles (metronidazole, tinidazole) totally inhibited *N. ceranae* proliferation, all other test substances were inactive. In summary, the assay proved suitable for substance screening and demonstrated the activity of two synthetic antibiotics against *N. ceranae*.

## Introduction

Wild and managed insect pollinators are crucial for the maintenance of natural ecosystems as well as for the agricultural production of many crops and fruit [1,2]. In most regions of the world, the supply with insect pollinators does not meet the steadily increasing demands in agriculture and it is this gap that makes the health of managed Western honey bee (*Apis mellifera*) colonies, which provide 90% of the commercial pollination in agriculture, a worldwide issue [3–5]. Hence, honey bee pests and pathogens became a research focus over the past decades and tremendous efforts to understand and combat honey bee diseases were undertaken in order to improve the health status of honey bee colonies and to avoid large scale colony losses. Reducing pathogen load and diseases in honey bees will not only help to reduce honey bee losses but might also have a positive impact on wild pollinators since evidence is accumulating that honey bee pathogens can spill over to bumblebees and might play a role in bumblebee decline [6,7]. Among the pathogens obviously threatening honey bees and wild bees alike is the microsporidian parasite *Nosema ceranae (N*. *ceranae)* [6,8,9].

Microsporidia are obligate intracellular parasites belonging to the phylum of fungi [10,11]. Honey bees are frequently found infected by *Nosema apis* (*N*. *apis*) and *N*. *ceranae* [12–15]. The metabolically inactive spores are the infectious form of *Nosema* spp. Once ingested by worker bees, e.g., in the course of cleansing activities inside the hive [16,17], the spores germinate in the midgut lumen thereby extruding their infection apparatus, the polar tube [18]. The polar tube penetrates the host cell membrane enabling the sporoplasm to be transferred into the host cell. Subsequently, the proliferation of the parasite in the midgut epithelial cells begins and characteristically spindle-shaped meronts, later sporonts, and finally new environmental spores are produced and released via cell lysis [19,20]. Infection causes a shortened life span in adult bees and changes in bee behavior; heavily infected bees might develop dysentery (nosemosis) which might lead to death of the individual bee but rarely of the entire colony [21–25]. While *N*. *apis* has long since been associated with the Western honey bee [14] and is well studied [12,26,27], *N*. *ceranae* is considered an emerging pathogen of the Western honey bee that has switched host from the Eastern honey bee (*Apis cerana*) only quite recently [13,28–34].

Virulence of *N*. *ceranae* for individual honey bees and impact of the disease on honey bee colonies is still controversially discussed [31,35–45]. However, despite this dispute it is well accepted that *N*. *ceranae* negatively affects honey bee health and does cause harm to honey bee colonies at least under certain conditions. For instance, evidence is accumulating that temperature plays a role in virulence and assertiveness of *N*. *ceranae* [13,42,46,47]. Hence, in the wake of climate changes, problems with colony losses due to *N*. *ceranae*, so far mainly reported from southern European countries like Spain [37,39], may extend to other regions too. An additional problem with *N*. *ceranae* infections of honey bee colonies just recently surfaced: the pathogen can spill over from honey bees into bumblebee populations causing fatal infections and contributing to bumblebee decline [6,8,9]. Therefore, *N*. *ceranae* poses a threat to managed and wild pollinators and developing treatment strategies or finding new agents active against *N*. *ceranae* should be a prime issue in contemporary bee research in order to save both honey bees and wild bees.

The only drug approved for *Nosema* control in honey bees is the antibiotic fumagillin derived from *Aspergillus fumigatus* which was and still is widely used to treat colonies infected with *N*. *apis* since the 1950s [48–52]. In Europe, the use of fumagillin against *Nosema* spp. is prohibited because the use of antibiotics in beekeeping practice is generally banned. However, in contrast to fumagillin’s efficacy against *N*. *apis*, *N*. *ceranae* might escape fumagillin control in honey bees [53] indicating that fumagillin may be unsuitable for the treatment of *N*. *ceranae* infections although previous reports showed that fumagillin treatment of *N*. *ceranae* infected colonies was effective [37,54]. It was even speculated that fumagillin may contribute to increased prevalence and pathogenicity of *N*. *ceranae* instead of being a curative measure against *N*. *ceranae* [53]. This situation makes it even more pressing to find new agents active against *N*. *ceranae*.

The quest for new drugs against honey bee pathogens has so far been performed via animal experiments using larvae, adult bees or entire colonies [55–58]. Such experiments are time consuming, difficult to standardize, and only feasible during the bee season when appropriate bee material is available. In addition, although using invertebrates in experiments does not pose any ethical problem, these experiments still are animal experiments and it is desirable to replace them. Cell culture based assays are a solution to these problems. They can be standardized by using a cell line. They are independent from the bee season and rather fast allowing testing thousands of substances per year. Promising candidate substances will still have to be tested *in vivo*, however, the number of bee experiments can be reduced to the absolute necessary minimum. Therefore, such an assay could provide a major progress in honey bee drug discovery and testing.

Here we present our results on establishing such an *in vitro* assay for medium throughput screening of substances with putative activity against *N*. *ceranae*. The assay is based on our recently developed cell culture model for *Nosema* spp., the lepidopteran cell line IPL-LD 65Y. This cell line originating from *Lymantria dispar* has been shown to be susceptible to *N*. *ceranae* infection and to support the entire life cycle of *N*. *ceranae* [20]. We combined the cell culture assay with quantitative detection of *N*. *ceranae* in infected cells via an RT-PCR-ELISA (reverse transcriptase-polymerase chain reaction-enzyme linked immunosorbent assay) protocol and adopted the assay for the 96-well microplate format to enable automated analysis. We first verified our test system using fumagillin as positive control, paromomycin as negative control, and clioquinol as a highly cytotoxic agent, to cover the possible range of effects on both cells and microsporidia. We then tested several commercially available agents for cytotoxicity and inhibition of *N*. *ceranae* intracellular development and demonstrate the efficacy of the synthetic antibiotics metronidazole and tinidazole against *N*. *ceranae*.

## Material and Methods

### Cell line and cell culture and agents

The IPL-LD 65Y cell line (*Lymantria dispar*) was obtained from the *Deutsche Sammlung von Mikroorganismen und Zellkulturen* (DSMZ, Braunschweig, Germany, No. ACC 181) and was maintained for routine culture in TC-100 medium (Lonza, Basel, Switzerland) with 11% fetal calf serum (FCS, PromoCell, Heidelberg, Germany). The cells were seeded with an initial concentration of 2E+05 cells per ml in tissue culture flasks (Greiner bio-one, Frickenhausen, Germany) and incubated at 27°C in a cooling incubator (Thermo Fisher Scientific, Schwerte, Germany). Cells were routinely passaged every seventh day.

Several commercially available substances were used in the screening assay. Fumagillin, surfactin, paromomycin, and metronidazole were obtained from Sigma-Aldrich (Taufkirchen, Germany); quinine, ornidazole, albendazole, tinidazole, clioquinol, and dimethylsulfoxid (DMSO) were obtained from VWR International (Darmstadt, Germany).

### MTT test for cell viability

Different concentrations of all substances were first tested for cytotoxicity. Fumagillin was tested within the range of 0.005 mg/ml to 0.100 mg/ml. All other substances were analyzed using concentrations between 0.02 mg/ml and 10 mg/ml. DMSO was used at a final concentration of 0.99% as solvent for all substances. Therefore, cytotoxicity of 0.99% DMSO for IPL-LD 65Y cells was also tested. Different working solutions of each substance were prepared to ensure that always only 1 μl had to be added to the cells in order to achieve the desired final concentrations.

For *in vitro*-cell viability tests a colorimetric assay (MTT test) was used. Cells were harvested at exponential growth phase, transferred to a 50 ml Falcon tube (VWR International, Darmstadt, Germany), and cell concentration was determined using a hemocytometer (Neubauer-Improved, VWR, Darmstadt, Germany). Cells were pelleted by centrifugation at 210 *g* for 10 min, the medium was aspirated, and fresh TC-100 cell culture medium, supplemented with 11% FCS, 250 μg/ml penicillin/streptomycin and 250 μl antibiotic/antimycotic-solution (Sigma Aldrich, Taufkirchen, Germany), was added to achieve a final cell density of 2.5E+05 cells/ml. 100 μl (2.5E+04 cells) were transferred into each well of a 96-well microplate. Subsequently, 1 μl of the appropriate working solution of each substance was added to the cells and cells were incubated for 72 hour at 27°C.

After 72 h, the microtiter plates were centrifuged at 210 *g* for 10 min to pellet the cells before the medium was carefully aspirated. Subsequently, 100 μl of 3-(4,5-dimethylthiazol-2-yl)-2,5-diphenyltetrazolium bromide (MTT, Carl Roth, Karlsruhe, Germany) at a concentration of 0.5 mg/ml TC-100 cell culture medium (supplemented with 11% FCS, 250 μg/ml penicillin/streptomycin and 250 μl antibiotic/antimycotic-solution) were added and cells were incubated for 3 h at 27°C. Viable cells convert the water soluble MTT into the water insoluble formazan. For formazan quantification, the cells in the microtiter plate were pelleted by centrifugation at 210*g* for 10 min. Subsequently, the cells were washed with 100 μl 1 x phosphate buffered saline (PBS) to remove residual medium. Cells were lysed and formazan was solubilized by adding 100 μl lysis-buffer (99.4% DMSO/0.6% acetic acid/10% SDS). Proportion of dead cells was analyzed at 595 nm excitation wavelength using an ELISA-reader (Synergy HT, BioTek, Bad Friedrichshall, Germany).

### Infection of IPL-LD 65Y cells and application of test substances

For isolation of *N*. *ceranae* spores, infected honey bees were identified via qualitative microscopic analysis and molecular species differentiation was performed essentially as already described [42,59]. Dissection of infected honey bees and isolation of *N*. *ceranae* spores from midguts for infection of cell cultures were performed as recently described [20]. Approximately 1E+08 freshly isolated *N*. *ceranae* spores were transferred into 1.5 ml reaction tubes (Eppendorf, Hamburg, Germany), dried in a vacuum concentrator (Eppendorf) for 30 min at 30°C, and immediately used for infection [20].

For infection, IPL-LD 65Y cells were harvested at exponential growth phase and pelleted via centrifugation at 210 *g* for 5 min. The cell pellet was washed twice with 1 ml of freshly prepared 0.1 M sucrose in 1xPBS and resuspended in sucrose buffer at a concentration of 2.5E+07 cells/ml. Immediately prior to infection, germination of the dried spores was initialized by resuspending the spores with 400 μl of 0.1 M sucrose in 1xPBS [42,60]. Germinating spores (1E+08) were resuspended in 100 μl cell suspension (2.5E+06 cells, resulting in a multiplicity of infection (MOI) of 40) and the cell-spore suspension was incubated for 5 min at room temperature. Infected cells were resuspended in 9.5 ml TC-100 cell culture medium supplemented with 11% FCS, 250 μg/ml penicillin/streptomycin, and 250 μl antibiotic/antimycotic-solution (Sigma Aldrich). Finally, 100 μl of the cell suspension (2.5E+04 infected cells) were carefully transferred into each well of a 96-well microplate (VWR).

For substance evaluation, 1 μl of each substance was added to the infected cells to achieve the desired final concentrations. Cells were incubated for 72 h at 27°C and, subsequently, infection status was determined independently both via PCR-ELISA and microscopic analysis. Each substance was tested in three independent replicates for both approaches.

### Molecular analysis of the infection status of IPL-LD 65Y cells via RT-PCR and RT-PCR-ELISA

For molecular analysis of the *N*. *ceranae* infection status of IPL-LD 65Y cells, the infected cells were transferred into a 1.5 ml PCR-clean reaction tube (Eppendorf) and stored at -70°C for further analysis. For RNA extraction, cell suspensions were resuspended in 300 μl RLT buffer (QIAGEN, Hilden, Germany) containing 3 μl β-mercaptoethanol (Roth). The cell homogenate was transferred to a QIAshredder tube (QIAGEN) and centrifuged at 16,100*g* for 4 min. The supernatant was gently mixed with 1 volume of 70% ethanol, transferred to an RNeasy spin column (QIAGEN), and centrifuged for 1 min at 8000*g*. The column was washed with 600 μl of RW1-buffer and again centrifuged for 30 sec at 8000*g*. To remove residual DNA, 80 μl of RNase-free DNase (QIAGEN) was added directly to the column, followed by incubation for 2 h at room temperature. The column was washed with 600 μl RW1-buffer and subsequent RNA extraction was performed essentially according to the manufacturer’s protocol.

For analyzing the gene expression pattern of several *N*. *ceranae*-genes in infected IPL-LD 65Y cells, primer pairs detecting *N*. *ceranae* genes were designed. To this end, the annotated protein sequences from cAMP-dependent protein kinase (acc. no. EQB61973.1), ruvb-like 1 DNA helicase (acc. no. EQB61817.1), chitin synthase d (acc. no. EQB60652.1), polar tube protein 2 (acc. no. EQB61988.1), and aqualysine peptidase (acc. no. EQB62240.1) of *N*. *apis*, spore wall protein 32 (acc. no. B3STN7.1) of *Nosema bombycis*, and checkpoint kinase (acc. no. AAL28053.1) of *Antonospora locustae* were blasted against hypothetical proteins of the *N*. *ceranae* shotgun genome sequences (acc. no. ACOL00000000.1) using the Basic Local Alignment Search Tool (BLAST, http://blast.ncbi.nlm.nih.gov/Blast.cgi). The identified nucleotide sequences showing the best homology to the blasted sequences were used for primer design (Table 1). Primers were designed using the online tool PrimerBLAST (http://www.ncbi.nlm.nih.gov/tools/primer-blast/). All primer sets were synthesized by Eurofins MWG Operon (http://www.eurofinsgenomics.eu/). For PCR-ELISA primer ptp2 fw (Table 1) was labeled with biotin and primer ptp2 rv (Table 1) was labeled with digoxigenin.

**Table 1:** Identification of hypothetical *N*. *ceranae* proteins based on annotated proteins from *N*. *apis*, *N*. *bombycis*, and *Antonospora locustae*.

annotated protein	*N*. *ceranae* protein acc. no.	primer names	sequence 5´→3´	bp
Nosema apis	XM_002995750.1	cAMP1 fw	-TCACCCTCTTTAATCAAAGTGACGCC-	318
cAMP-dep. protein kinase EQB61973.1		cAMP1 rv	-CGATGAAGCCCTGCTCTTGTCTC-	
Nosema apis	XM_002995407.1	hel fw	-CGATATGCACAACGTCTCCCACTT-	323
ruvb-like 1 DNA helicase EQB61817.1		hel rv	-TCCTAAGGGATGTGGTAAAACCGCC-	
*Antonospora locustae* checkpoint protein kinase AAL28053.1	XM_002995655.1	chk1 fw	-TGGCTCCTGAGGTTGCAATGGAAA-	303
		chk1 rv	-GTGTATCCCTGGCCACAGAACTTTCT-	
Nosema apis	XM_002996666.1	chs fw	-GAGTTGCTTTCCAAAGGCTCGCT-	278
chitin synthase d EQB60652.1		chs rv	-TGCGAAATGCATGGCGAGAGA-	
Nosema apis	XM_002995446.1	ptp2 fw	-TGCTTTTTAGGTGGCAACTTGGCT-	269
polar tube protein 2 EQB61988.1		ptp2 rv	-GCTTTTGTAGGATCTGTTCCCGGCA-	
Nosema bombycis	XM_002996303.1	swp32 fw	-GCTGAAGGAATACCCGAGAAGCTGC-	531
spore wall protein 32 B3STN7.1		swp32 rv	-AGGTAGACACTTTGGGATGCGGGA-	
Nosema apis	XM_002996635.1	ep fw	-GCTGCAACCATAGCAGGCGC-	303
aqualysine peptidase EQB62240.1		ep rv	-AGTATCAATCACATAATCCCTTCTC-	

Identified nucleic acid sequences were used for primer design (PrimerBLAST).

One-step RT-PCR reactions were performed according to standard protocols (One-step-RT-PCR kit, QIAGEN) using a reaction volume of 25 μl, 20 ng RNA per reaction, and the following temperature scheme: 30 min at 50°C, 15 min at 95°C followed by 35 cycles with 1 min at 94°C, 1 min at 58°C (optimal for all primer sets), 1 min at 72°C, each, including a final elongation step for 10 min at 72°C. PCR reaction mixes were stored at 4°C until further analysis. Reaction products obtained with unlabeled primers were analyzed via agarose gel electrophoresis, stained with ethidium bromide, and visualized under UV-light. Reaction products obtained using biotin- and digoxigenin-labeled *ptp*2-primers were quantified via PCR-ELISA.

For quantification of *N*. *ceranae* in infected cells, a recently published protocol for the quantification of *Leishmania* parasites in host tissues [61] was modified. Streptavidin-coated 96-well microplates (VWR) were washed four times with ELISA-washing buffer (0.5% Tween 20 in 1xPBS). 1 μl of the RT-PCR reaction mix obtained by using *ptp*2 primers labeled with biotin and digoxigenin (see above) was resuspended with 99 μl of 2% FCS in 1xPBS and transferred into the wells. After incubation for one hour, the wells were washed six times with ELISA-washing buffer and incubated for 45 min with 100 μl anti-digoxigenin-peroxidase fab-fragments (Roche Diagnostics, Mannheim, Germany, 0.15 U/ml) in 2% FCS/PBS-buffer. The wells were washed eight times with ELISA-washing buffer and dried at room temperature. 100 μl of the peroxidase substrate 2,2'-Azino-di-[3-ethylbenzthiazoline sulfonate (6)] diammonium salt (ABTS; Roche Diagnostics) at a concentration of 0.3 mg/ml ABTS in 100 mM citric acid (Carl Roth, pH 4.35) with 0.1% H_2_O_2_ (Carl Roth) were added to each well and cells were incubated in the dark to develop color reaction. The color reaction was stopped after 5 min by adding 50 μl of 500 mM oxalic acid (Carl Roth). Arbitrary units for gene expression of the *N*. *ceranae* polar tube protein were obtained by reading the absorbance values of the plates at 405 nm excitation wavelength with 620 nm reference wavelength using an ELISA-reader. Arbitrary units of gene expression were compared and statistically analyzed by ANOVA with Kruskal-Wallis nonparametric multiple comparison test with α<0.05 using the GraphPad Prism 6.01 software package. Statistical differences were classified: not significantly different: n.s., p ≥ 0.05; significantly different: *, 0.05 > p ≥ 0.01; **, 0.01 > p ≥ 0.001.

### Microscopic analysis of the infection status of IPL-LD 65Y cells

For microscopic analysis of the *N*. *ceranae* infection status of IPL-LD 65Y cells, the infected cells were centrifuged onto glass slides (VWR) with a cell spin (Tharmac, Waldsolms, Germany) at 60 *g* for 5 min. The cells were stained with Giemsa and microscopically analyzed essentially as already described [20].

## Results and Discussion

### Development of an *in vitro* screening assay for candidate agents against *N*. *ceranae*

**Fig 1:**
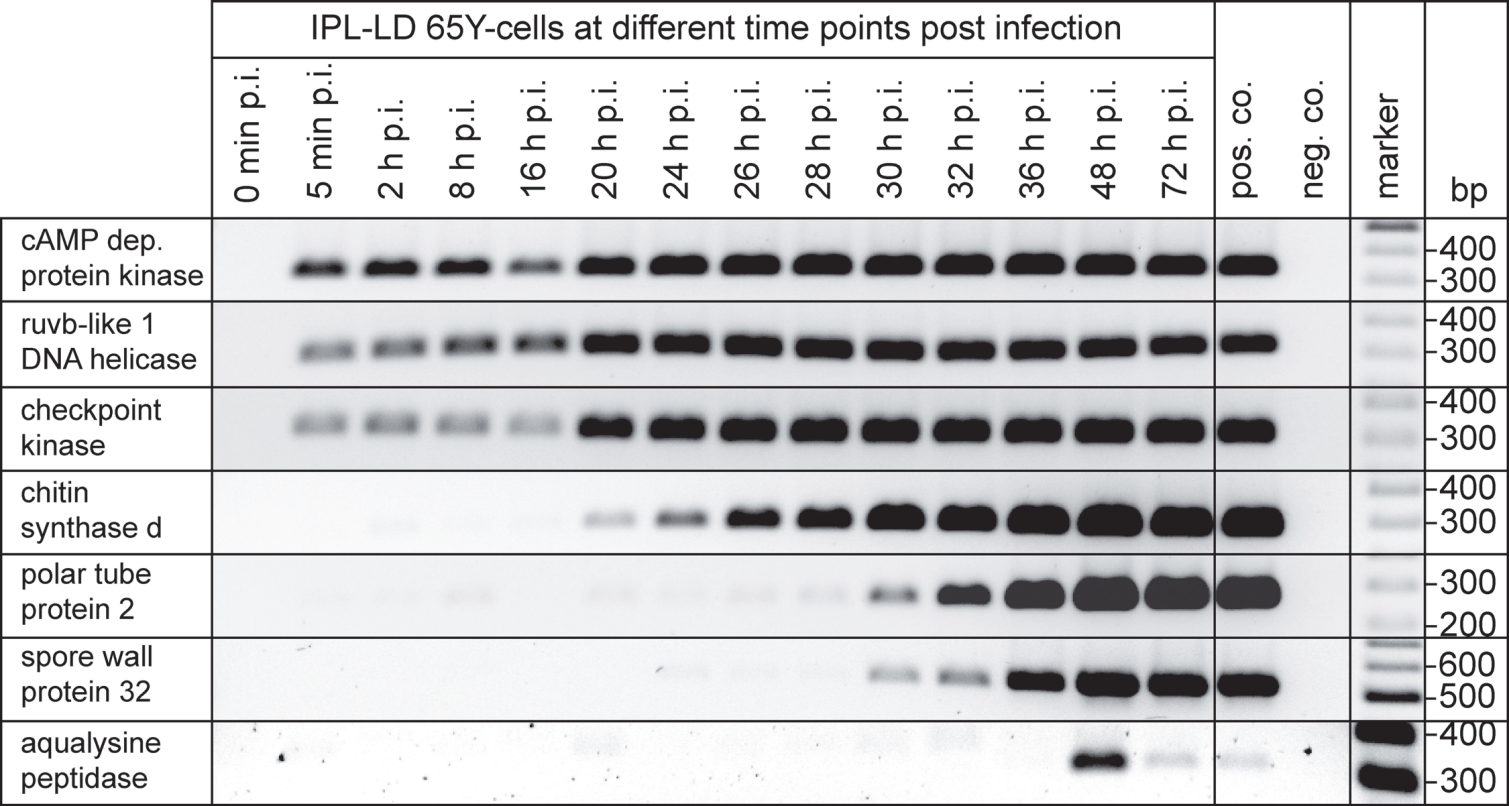
*Nosema ceranae* gene expression pattern in *N*. *ceranae* in infected IPL-LD 65Y cells analyzed over 72 h post infection. IPL-LD 65Y cells were infected with *N*. *ceranae* and RNA was extracted at different time points post infection (0 h–72 h p.i.). Time course of expression of different genes was analyzed via RT-PCR using the primers given in Table 1.

The availability of a cell culture model for *Nosema* spp. infections [20] opened the possibility of developing a cell culture-based screening assay for substances active against *Nosema* spp. or specifically against *N*. *ceranae*. For such an assay, quantification of *N*. *ceranae* infection is a prerequisite. To this end we adapted a recently published PCR-ELISA protocol, which had originally been developed for detection and quantification of *Leishmania* parasites in host tissues [61], to *N*. *ceranae*. The described PCR-ELISA relies on the use of one biotin- and one digoxigenin-labeled primer per PCR-product and on the detection of the PCR products by sandwich ELISA. The PCR products are bound to streptavidin coated microplates by virtue of their incorporated biotin and they are visualized by anti-digoxigenin antibodies binding to the incorporated digoxigenin. Primer molecules that are not part of the newly generated PCR products will not produce any background because they are not detected by this ELISA. A prerequisite for establishing an RT-PCR-ELISA for the detection and quantification of *N*. *ceranae* was the selection of appropriate primers detecting genes expressed during infection. Previous studies had shown that 72 hours after infection merogony is nearly completed and sporogony, i.e. the development of new spores marking the final phase of the vegetative cycle, is initiated [20]. Therefore, we first analyzed the expression pattern of several *N*. *ceranae* genes during infection of IPL-LD 65Y cells via RT-PCR over 72 hours (Fig. 1). Primers (Table 1) were designed on the basis of the published *N*. *ceranae* genome sequence data [62]. Expression of cAMP-dependent protein kinase, ruvb-like 1 DNA helicase, and checkpoint kinase was detectable already five minutes post infection and throughout the entire observation period. In contrast, expression of chitin synthase d, polar tube protein 2 (PTP2), and spore wall protein 32 (SWP32) started around 20 hours post infection or even later and was detectable until the end of the observation period. Expression of aqualysine peptidase could only be detected during a narrow time window around 48 hours post infection.

Based on these results we chose expression of *ptp*2 as measure of *N*. *ceranae* infection in cultured cells because detection of *ptp*2 expression seemed suitable as indicator for successful development of *N*. *ceranae* in infected cells whereas absence of *ptp*2 expression should be indicative for non-infected cells. To evaluate our assay, IPL-LD 65Y cells were infected with *N*. *ceranae* and either left untreated or immediately treated with DMSO (0.99%) which was used as solvent for all substances, two control substances, paromomycin (final concentration of 10 mg/ml) as negative control, fumagillin (final concentration of 0.01 mg/ml) as positive control, and clioquinol (final concentration 0.1 mg/ml) as cytotoxic agent. Paromoycin has been shown to be ineffective against microsporidia because these parasites lack an essential binding site for this drug [63] although one historical study suggested a possible effect of paromomycin on *N*. *apis* infection in honey bees [64]. Fumagillin has been demonstrated to be active against *N*. *apis* and has been widely used to control nosemosis in bee colonies [48,50–52]. Clioquinol is a chelating agent for copper, zink, and iron with cytotoxic activity [65,66] and, therefore, was used as control to demonstrate the robustness of the assay even for cases when test substances applied to the infected cells turn out to be cytotoxic.

First we tested the effect of the test substances on the viability of IPL-LD 65Y cells via MTT tests. DMSO (0.99%) serving as solvent for all substances had no cytotoxic effect on the cells (Table 2). Fumagillin was cytotoxic when used at concentrations of 0.1 mg/ml and 0.05 mg/ml but showed no cytotoxicity at 0.01 mg/ml and 0.005 mg/ml (Table 2). Therefore, we used 0.01 mg/ml fumagillin in our *in vitro*-infection assay. Paromomycin had no cytotoxic activity on IPL-LD 65Y cells in the concentrations tested while the cytotoxicity of clioquinol for IPL-LD 65Y cells was evident at both 1 mg/ml and 0.1 mg/ml (Table 2).

**Table 2:** Cytotoxicity for IPL-LD 65Y cells of different agents used for establishing and testing the *in vitro* screening assay.

agent	final concentration	cytotoxic effect on cultured cells [proportion of dead cells in %]
w/o	-	0
fumagillin	0.005 mg/ml	0
fumagillin	0.01 mg/ml	0
fumagillin	0.05 mg/ml	65
fumagillin	0.1 mg/ml	78
paromomycin	1.0 mg/ml	0
paromomycin	10 mg/ml	0
clioquinol	0.1 mg/ml	86
clioquinol	1.0 mg/ml	84
DMSO	0.99%	0
albendazole	0.1 mg/ml	0
albendazole	1 mg/ml	28
metronidazole	0.2 mg/ml	0
metronidazole	2 mg/ml	7
tinidazole	0.2 mg/ml	0
tinidazole	2 mg/ml	19
ornidazole	1 mg/ml	0
ornidazole	10 mg/ml	89
surfactin	0.02 mg/ml	35
surfactin	0.2 mg/ml	89
quinine	0.2 mg/ml	14
quinine	2 mg/ml	88

**Fig 2:**
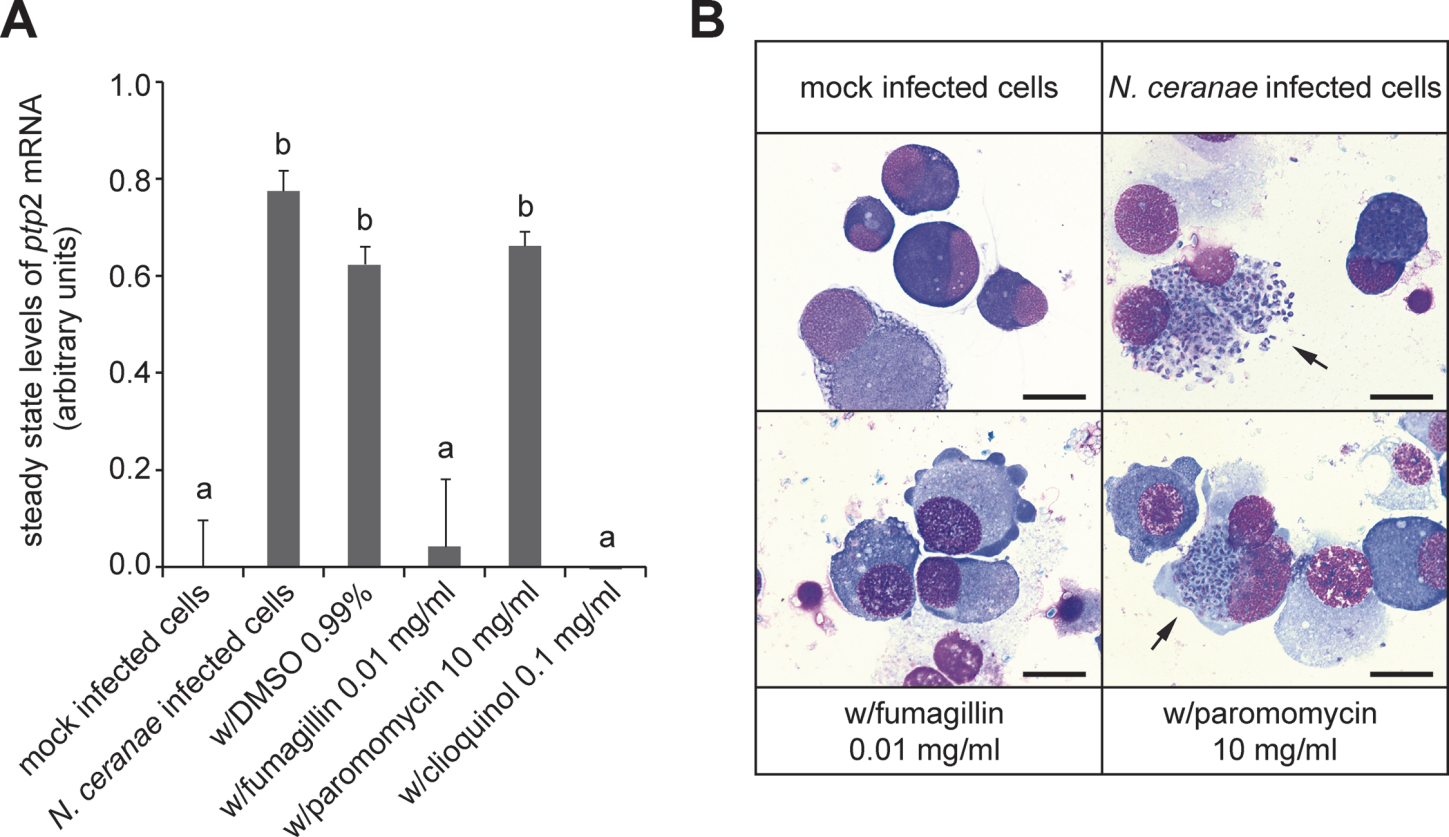
Evaluation of the suitability of a novel RT-PCR-ELISA-based medium throughput screening assay for determining the infection status of *N*. *ceranae* infected IPL-LD 65Y cells. (A) Expression levels of the *N*. *ceranae ptp*2 gene in infected IPL-LD 65Y cells were determined via an RT-PCR-ELISA protocol. Cells were infected with *N*. *ceranae* and left untreated or were directly treated with DMSO (0.99%), fumagillin (0.01 mg/ml), paromomycin (10 mg/ml), or clioquinol (0.1 mg/ml). Mock infected cells served as control. RNA was extracted at 72 h post infection. Results of the RT-PCR-ELISA for steady state levels of *ptp*2 mRNA were expressed in arbitrary units. All columns represent mean values ± SD of three independent replicates per group. Columns with different letters differ significantly, while columns with the same letter are not significantly different (ANOVA with Kruskal-Wallis nonparametric multiple comparison test with α<0.05). (B) Microscopic analysis of infected IPL-LD 65Y cells for the presence or absence of intracellular stages of *N*. *ceranae at* 72 h post infection was used to confirm all results obtained via the RT-PCR-ELISA protocol. Shown are mock-infected cells, *N*. *ceranae*-infected cells and infected cells treated with fumagillin (0.01 mg/ml) or paromomycin (10 mg/ml). Arrows point to cells filled with spore stages of *N*. *ceranae*. Scale bars represent 25 μm.

Analysis of *ptp*2 mRNA expression at 72 hours post-infection using the RT-PCR-ELISA protocol demonstrated expression of *ptp*2 in infected, untreated cells while no *ptp*2 expression (0 ± 0.1 arbitrary units) was detectable in mock infected control cells (Fig. 2A). This difference was highly significant (p-value < 0.0001). The slight decrease in *ptp*2-expression in infected, DMSO-treated cells was not significant compared to infected, non-treated cells (p-value > 0.9999). Infected cells treated with fumagillin showed only residual *ptp*2 expression not significantly different (p-value = 0.9999) from mock infected control cells (Fig. 2A) consistent with the approved efficacy of fumagillin in the treatment of *N*. *ceranae* infections [48]. Treatment of infected cells with paromomycin resulted in *ptp*2 mRNA steady state levels which did not differ significantly (p-value = 0.9999) from both infected, untreated cells and infected, DMSO-treated cells (Fig. 2A) confirming that paromomycin indeed has no activity against *N*. *ceranae*. In infected cells treated with clioquinol, no *ptp*2 expression was detectable (Fig. 2A) as a result of the cytotoxic activity of the substance which killed nearly 90% of the cells (Table 2).

Microscopic analysis of Giemsa-stained cells for presence or absence of intracellular stages of *N*. *ceranae* visually confirmed the results obtained via the RT-PCR-ELISA protocol (Fig. 2B). Mock infected control cells as well as *N*. *ceranae*-infected, fumagillin treated cells did not contain any vegetative or spore stages of *N*. *ceranae*. In contrast, *N*. *ceranae* infected cells as well as infected cells treated with paromomycin were filled with blue-stained spore stages of *N*. *ceranae* as expected at 72 hours post infection [20]. In the few cells still viable after clioquinol treatment no vegetative stages of *N*. *ceranae* were detectable consistent with the lack of detectable *ptp*2 mRNA expression.

These results indicated that *N*. *ceranae* infected IPL-LD 65Y cell cultures combined with an RT-PCR-ELISA protocol for the quantitative detection of *ptp*2 expression constitutes a well suited assay for the identification of novel drugs with *in vitro* activity against *N*. *ceranae*. The 96-well-format of the RT-PCR-ELISA will allow medium throughput screening of substances thus providing a state-of-the-art tool for anti-nosemosis drug discovery.

### Identification of substances active against *N*. *ceranae* through the novel *in vitro* screening assay

**Fig 3:**
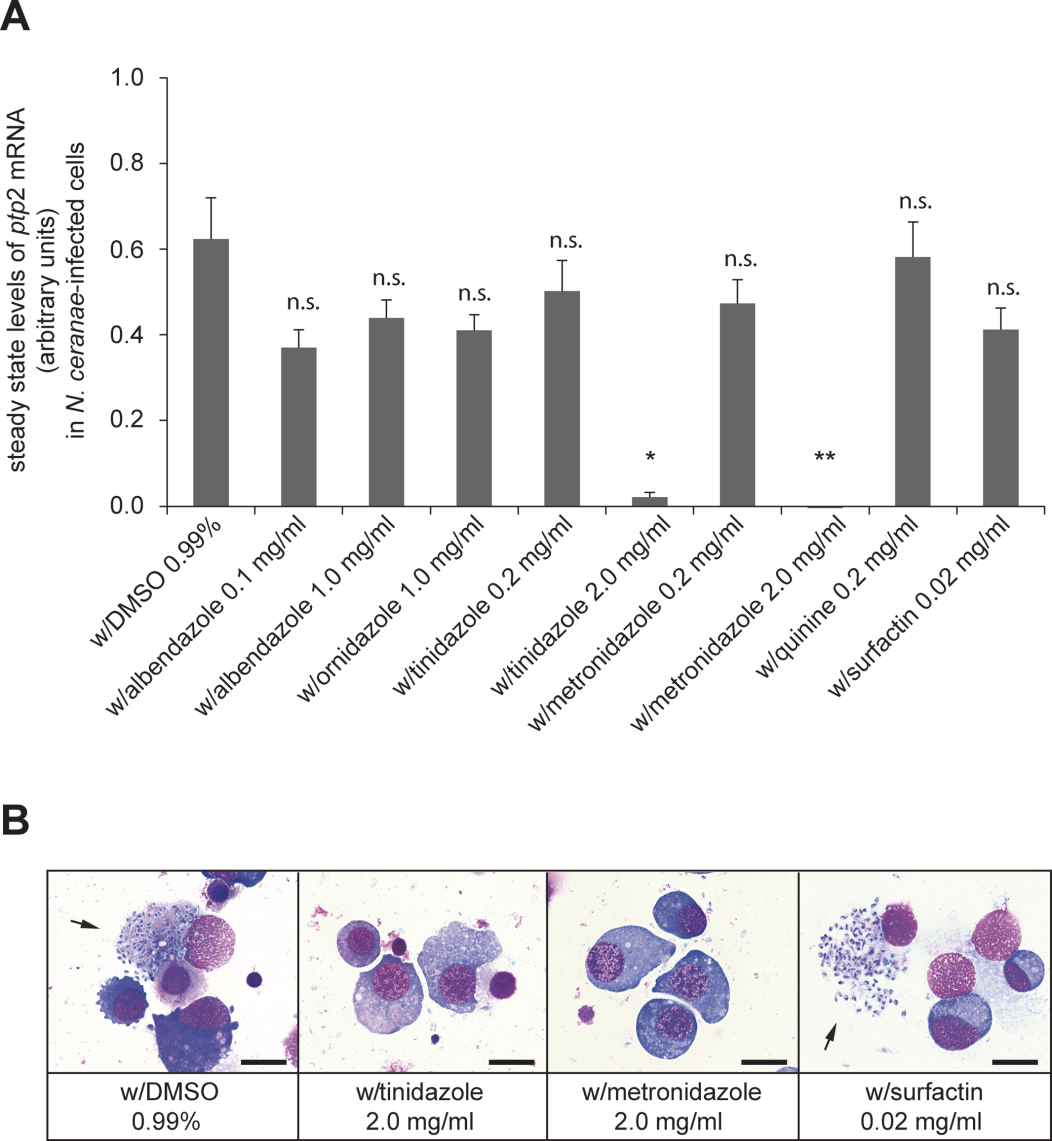
Effect of different substances with putative activity against *N*. *ceranae* infections in cultured IPL-LD 65Y cells. (A) Expression levels of the *N*. *ceranae ptp*2 gene in infected IPL-LD 65Y cells were determined via an RT-PCR-ELISA protocol. Cells were infected with *N*. *ceranae* and then directly treated with DSMO, albendazole, ornidazole, tinidazole, metronidazole, quinine, and surfactin in the given concentrations. DMSO-treated, infected cells served as control and reference for evaluating the effect of the tested substances. RNA was extracted at 72 h post infection. Results of the RT-PCR-ELISA for steady state levels of *ptp*2 mRNA were expressed in arbitrary units. All columns represent mean values ± SD of three independent replicates per group. Statistical analysis was performed with ANOVA and Kruskal-Wallis nonparametric multiple comparison test (not significantly different: n.s., p ≥ 0.05; significantly different: *, 0.05 > p ≥ 0.01; **, 0.01 > p ≥ 0.001. (B) Microscopic analysis of infected IPL-LD 65Y cells for the presence or absence of intracellular stages of *N*. *ceranae at* 72 h post infection to visualize the effect of different substances on *N*. *ceranae* infection in cultured cells. Results obtained with DMSO (0.99%), tinidazole (2.0 mg/ml), metronidazole (2.0 mg/ml), and surfactin (0.02 mg/ml) are shown. Arrows point to cells filled with spore stages of *N*. *ceranae*. Scale bars represent 25 μm.

We next used our newly established screening assay and tested several antimicrobial agents for their activity against *N*. *ceranae* in infected cell cultures aiming at identifying novel agents with anti-nosemosis activity comparable to that of fumagillin. The substances to be tested were selected based on their known inhibitory effect on other microsporidia, fungi or intracellular parasites. We tested the synthetic antibiotics albendazole, ornidazole, tinidazole, and metronidazole because both *in vitro* and *in vivo* studies have demonstrated that benzimidazoles and nitroimidazoles and its derivatives have activity against microsporidia in vertebrates as well as in invertebrates [67–71]. Furthermore, we tested quinine, which was found to be effective against *Encephalitozoon* species in grasshoppers [72], and surfactin because it was shown to influence *N*. *ceranae* development when fed to infected honey bees [55]. For all agents, the effect on cell viability (MTT test) and the activity against *N*. *ceranae* infection (cell culture based assay) were evaluated. DMSO was used as solvent for all agents and, hence, infected cells treated with DMSO served as control and reference for all tested substances (Fig. 3A). DMSO in the tested final concentration of 0.99% was neither cytotoxic (Table 2) nor active against *N*. *ceranae* (Figs. 2A and 3B) and thus did not influence the analysis of the test substances.

Albendazole was applied to uninfected cells with final concentrations of 0.1 mg/ml and 1.0 mg/ml. At the higher concentration, MTT tests revealed 28% dead cells indicating some cytotoxic activity at this concentration. No cytotoxicity was observed when 0.1 mg/ml albendazole were applied (Table 2). Despite a ten-fold difference in albendazole concentration, the *ptp*2 mRNA steady state levels were not significantly different (p-value > 0.9999) between the two treatments. When compared to infected, DMSO-treated cells, the reduction in *ptp*2 gene expression was not significant for both concentrations (p-value = 0.4818 for 0.1 mg/ml and p-value > 0.9999 for 1 mg/ml) suggesting a still ongoing infection (Fig. 3A). Microscopic analysis of Giemsa-stained cells confirmed this result and revealed numerous infected cells filled with blue stained spore stages (data not shown). Therefore, it was not possible to cure infected cells with non-cytotoxic concentrations of albendazole (Fig. 2A-B).

This lack of *in vitro* activity of albendazole against *N*. *ceranae* is in contrast to its activity against *N*. *bombycis* both *in vitro* and *in vivo*. In *Spodoptera frugiperda* cells experimentally infected with *N*. *bombycis*, a final concentration of as little as 0.0053 mg/ml albendazole already caused 57.7% reduction in the proportion of infected cells (40.7% reduced to 26.9%; [69]). *In vivo* assays performed with 2.5 mg/ml, 5.0 mg/ml, and 10 mg/ml albendazole fed to *N*. *bombycis* infected silkworm larvae (*Bombyx mori*) revealed 86%, 96%, and 100% disease reduction, respectively [70]. Hence, *N*. *bombycis* in cell culture is highly sensitive towards albendazole. However, 470- to 2000-fold higher concentrations were needed for effective disease reduction *in vivo*.

We next tested the nitroimidazoles ornidazole, tinidazole, and metronidazole which had also shown *in vivo* activity against *N*. *bombycis* in silkworm larvae and resulted in 80% to 90% disease reduction when orally applied at 2.5 mg/ml to 10 mg/ml, respectively [70]. In our *in vitro* cell culture assay, ornidazole was highly toxic for IPL-LD 65Y cells (89% dead cells) when applied at 10 mg/ml (Table 2). When used at 1 mg/ml, a non-cytotoxic concentration (Table 2), no significant reduction in *ptp*2 mRNA steady state levels compared to infected cells (p-value > 0.9999; Fig. 3A) was observed. Accordingly, no obvious effect against *N*. *ceranae* was observed in Giemsa-stained cells (data not shown). Therefore, ornidazole like albendazole has no significant *in vitro* activity against *N*. *ceranae*.

Tinidazole and metronidazole were weakly cytotoxic at 2 mg/ml with 19% and 7% dead IPL-LD 65Y cells, respectively. All cells survived when 0.2 mg/ml were applied (Table 2). Obviously, cytotoxicity of metronidazole is highly dependent on the cells used because cell viability of uninfected vertebrate cells (Madin-Darby Canine Kidney epithelial (MDCK) cells) exposed to 0.050 mg/ml metronidazole was already reduced to 52% [67]. Steady state levels of *ptp*2 mRNA were not significantly reduced in infected IPL-LD 65Y cells by 0.2 mg/ml tinidazole (p-value > 0.9999) or metronidazole (p-value > 0.9999) compared to DMSO-treated infected cells (Fig. 3A). However, when tinidazole and metronidazole were used at concentrations of 2 mg/ml, *ptp2* mRNA expression was no longer detected (Fig. 3A). Steady state levels of *ptp*2 mRNA were significantly different between DMSO-treated, infected cells and metronidazole- or tinidazole-treated, infected cells (p-value = 0.007 or p-value = 0.0377, respectively) (Fig. 3A). In addition, there was no statistically significant difference between mock infected cells and metronidazole- or tinidazole-treated, infected cells (p-value > 0.9999 or p-value > 0.999, respectively) (data not shown). In accordance with these molecular data (Fig. 3A), no single infected cell could be observed by microscopic analysis of Giemsa-stained cells (Fig. 3B). These results indicated that at a concentration of 2 mg/ml both tinidazole and metronidazole can completely inhibit *N*. *ceranae* infection of IPL-LD 65Y cells and, therefore, were as effective as fumagillin *in vitro*. However, both substances will hardly have a future as anti-nosemosis drugs in honey bees. Nitroimidazoles are antibiotics and the use of antibiotics in honey bee colonies and in particular the use of nitroimidazoles in food animals and animal food is prohibited in many countries [73] including the European Union (Commission Regulation (EU) No 37/2010 of 22 December 2009).

Next, we evaluated the *in vitro* activity of quinine against *N*. *ceranae*. Tested at 2 mg/ml, quinine was highly cytotoxic for IPL-LD 65Y cells (88% dead cells, Table 2). Reducing the final quinine concentration to 0.2 mg/ml resulted in a markedly reduced cytotoxicity with only 14% dead cells (Table 2). However, there was no significant difference in *ptp*2 mRNA steady state levels between quinine-treated and DMSO-treated infected cells (p-value > 0.9999) indicating that quinine had no effect on *N*. *ceranae* infection in cell culture (Fig. 3A). This was not entirely in contrast to results obtained with of *Encephalitozoon* sp. in infected grasshoppers. While intra-hemocelic injection of quinine resulted in spore counts of about 0.11 x 10^6^ spores/mg tissue/grasshoppers which were significantly different from control (0.4 x 10^6^/mg tissue/grasshopper; [72]), oral treatment with about 1.68 mg quinine/day had resulted in a non-significant 53% reduction in spores [74]. Hence, quinine seemed not to be generally active against *Encephalitozoon* sp. in grashoppers but only under certain conditions whereupon intra-hemocelic injection is rather artificial and the more workable oral application did not result in a significant disease reduction.

Another promising anti-nosemosis agent was the bacterial peptide secondary metabolite surfactin because of its reported effect on *N*. *ceranae* development in infected bees [55]. However, surfactin is able to lyse cells and, hence, a drawback of surfactin is its non-specific cytotoxicity which has already been demonstrated in several vertebrate cell lines [75]. Concentrations of less than 0.025 mg/ml had no effect on vertebrate cell viability but when exposed to concentrations greater than 0.073 mg/ml no cells survived. Testing the cytotoxicity of surfactin for IPL-LD 65Y cells revealed that applying 0.2 mg/ml surfactin already resulted in 90% dead cells and even with 0.02 mg/ml still 35% of the cells died (Table 2). These results suggested that invertebrate cells are as sensitive or even more sensitive towards surfactin than vertebrate cells. When the lower surfactin concentration of 0.02 mg/ml was tested for its activity against *N*. *ceranae* in infected cells, we observed no significant reduction in *ptp*2 mRNA steady state levels in comparison to DMSO-treated infected cells (p-value > 0.9999) (Fig. 3A). This was substantiated by microscopic analysis of Giemsa-stained cells which revealed numerous infected cells filled with blue stained spore stages despite surfactin treatment (Fig. 3B).

These results were contradictory to the published effect of two different surfactin preparations (S1 and S2) produced by different strains of *Bacillus subtilis* on *N*. *ceranae* infection in honey bees [55]. It was reported that 5 mg/ml surfactin supplied *ad libitum* before and after *N*. *ceranae* infection resulted in an increased proportion of bees exhibiting low (0–1E+06) spore intensity and a decreased proportion of bees with high (>1E+07) spore intensity when compared to untreated bees. However, the effect was significant only for the S2-surfactin preparation. In addition, this S2-surfactin preparation also had a significant effect on spore viability because incubation of spores for 40 hours with 10 mg/ml S2-surfactin resulted in an increased proportion of bees exhibiting medium (1E+06–1E+07) intensity of spores in the midgut when compared to untreated bees (23.2% to 58.8%) while at the same time the percentage of bees with high (>1E+07) spore intensity decreased from 63.2% to 23.5%. However, the proportion of bees with low (0–1E+06) spore intensity did not change upon treatment. These results are remarkable in that they suggest that there is no general effect of surfactin on *N*. *ceranae* infections in bees but the effect is dependent on the surfactin preparation used. This might be due to the fact that the cyclic lipopeptide surfactin produced by *B*. *subtilis* has been shown to be a mixture of isoforms as a result of amino acid substitutions in the peptide ring and variations in the chain length and branching of its hydroxy fatty acid component [76,77]. These isoforms are also reported to have slightly different properties [77–80] which might explain the differences in efficacy between different surfactin preparations observed in infected bees [55]. Hence, it is conceivable that the commercially obtained surfactin used in our cell culture assay is a less active isoform like the S1-surfactin preparation used by Porrini and co-workers [55]. A more thorough analysis of surfactin activity against *N*. *ceranae* including the identification of the structural characteristics correlating with anti-nosemosis activity and the assessment of cytotoxicity of orally administered surfactin in honey bees is necessary to finally decide on the applicability of surfactin as anti-nosemosis drug in honey bees.

## Conclusion

We here present a screening assay for the identification of substances exhibiting activity against *N*. *ceranae*, which is based on *N*. *ceranae* infected cultured cells and coupled with an RT-PCR-ELISA protocol for determining *N*. *ceranae* infection intensity. This novel screening assay can also be performed with *N*. *apis* infected IPL-LD 65Y-cell cultures [20] and, hence, provides a so far unavailable screening platform to test many substances at a time for their potential anti-nosemosis activity. Laborious and time-restricted animal experiments and *in vivo*-tests will be reduced to a minimum because only promising candidates will be tested in bees and in the field. With the help of this cell culture based medium throughput screening system, the analysis or development of new anti-nosemosis substances will be accelerated and simplified in the future.

## References

[1] Garibaldi LA, Steffan-Dewenter I, Winfree R, Aizen MA, Bommarco R, et al (2013) Wild pollinators enhance fruit set of crops regardless of honey bee abundance. Science 339: 1608–1611. 10.1126/science.1230200 23449997

[2] Aizen MA, Garibaldi1 LA, Cunningham SA, Klein AM (2009) How much does agriculture depend on pollinators? Lessons from long-term trends in crop production. Ann Bot (Lond) 103: 1579–1588. 10.1093/aob/mcp076 19339297 PMC2701761

[3] Aizen MA, Garibaldi LA, Cunningham SA, Klein AM (2008) Long-term global trends in crop yield and production reveal no current pollination shortage but increasing pollinator dependency. Curr Biol 18: 1572–1575. 10.1016/j.cub.2008.08.066 18926704

[4] Aizen MA, Harder LD (2009) The global stock of domesticated honey bees is growing slower than agricultural demand for pollination. Curr Biol 19: 1–4. 10.1016/j.cub.2008.11.058 19427214

[5] Klein A-M, Vaissiere BE, Cane JH, Steffan-Dewenter I, Cunningham SA, et al (2007) Importance of pollinators in changing landscapes for world crops. Proc R Soc B 274: 303–313. 17164193 10.1098/rspb.2006.3721 PMC1702377

[6] Plischuk S, Martín-Hernández R, Prieto L, Lucía M, Botías C, et al (2009) South American native bumblebees (Hymenoptera: Apidae) infected by *Nosema ceranae* (Microsporidia), an emerging pathogen of honeybees (*Apis mellifera*). Environ Microbiol Rep 1: 131–135. 10.1111/j.1758-2229.2009.00018.x 23765744

[7] Genersch E, Yue C, Fries I, de Miranda JR (2006) Detection of *Deformed wing virus*, a honey bee viral pathogen, in bumble bees (*Bombus terrestris* and *Bombus pascuorum*) with wing deformities. J Invertebr Pathol 91: 61–63. 16300785 10.1016/j.jip.2005.10.002

[8] Graystock P, Yates K, Darvill B, Goulson D, Hughes WOH (2013) Emerging dangers: Deadly effects of an emergent parasite in a new pollinator host. J Invertebr Pathol 114: 114–119. 10.1016/j.jip.2013.06.005 23816821

[9] Fürst MA, McMahon DP, Osborne JL, Paxton RJ, Brown MJF (2014) Disease associations between honey bees and bumblebees as a threat to wild pollinators. Nature 506: 364–366. 10.1038/nature12977 24553241 PMC3985068

[10] Corradi N, Keeling PJ (2009) Microsporidia: a journey through radical taxonomical revisions. Fungal Biol Rev 23: 1–8.

[11] Lee SC, Corradi N, Byrnes III EJ, Torres-Martinez S, Dietrich FS, et al (2008) Microsporidia evolved from ancestral sexual fungi. Curr Biol 18: 1675–1679. 10.1016/j.cub.2008.09.030 18976912 PMC2654606

[12] Fries I (1988) Infectivity and multiplication of *Nosema apis* Z. in the ventriculus of the honey bee. Apidologie 19: 319–328.

[13] Fries I, Martin-Hernandez R, Meana A, Garcia-Palencia P, Higes M (2006) Natural infections of *Nosema ceranae* in European honey bees. J Apicult Res 45: 230–233.

[14] Zander E (1909) Tierische Parasiten als Krankheitserreger bei der Biene. Münchener Bienenzeitung 31: 196–204. 10.1016/j.bbagen.2015.01.008 25603559

[15] Fries I, Feng F, daSilva A, Slemenda SB, Pieniazek NJ (1996) *Nosema ceranae* n sp (Microspora, Nosematidae), morphological and molecular characterization of a microsporidian parasite of the Asian honey bee *Apis cerana* (Hymenoptera, Apidae). Eur J Protistol 32: 356–365.

[16] Bailey L (1955) The epidemiology and control of Nosema disease of the honey bee. Ann Appl Biol 43: 379–389.

[17] Bailey L (1955) The infection of the ventriculus of the adult honeybee by *Nosema apis* (Zander). Parasitology 45: 86–94. 14370833 10.1017/s0031182000027451

[18] Bigliardi E, Sacchi L (2001) Cell biology and invasion of the microsporidia. Microbes Infect 3: 373–379. 11369274 10.1016/s1286-4579(01)01393-4

[19] Cali A, Takvorian PM (1999) Developmental morphology and life cycles of the microsporidia In: Wittner M, Weiss LM, editors. The microsporidia and microsporidiosis. Washington, D.C.: ASM Press pp. 85–128.

[20] Gisder S, Möckel N, Linde A, Genersch E (2011) A cell culture model for *Nosema ceranae* and *Nosema apis* allows new insights into the life cycle of these important honey bee pathogenic microsporidia. Environ Microbiol 13: 404–413. 10.1111/j.1462-2920.2010.02346.x 20880328

[21] Anderson DL, Giacon H (1992) Reduced pollen collection by honeybee (Hymenoptera, Apidae) colonies infected with *Nosema apis* and sacbrood virus. J Econ Entomol 85: 47–51.

[22] Fries I, Ekbohm G, Villumstad E (1984) *Nosema apis*, sampling techniques and honey yield. J Apicult Res 23: 102–105.

[23] Kralj J, Fuchs S (2010) *Nosema* sp. influences flight behavior of infected honey bee (*Apis mellifera*) foragers. Apidologie 41: 21–28.

[24] Wang D-I, Moeller FE (1969) Histological comparisons of the development of the hypopharyngeal glands in healthy and *Nosema*-infected worker honey bees. J Invertebr Pathol 14: 135–142.

[25] Wang D-I, Moeller FE (1970) The division of labor and queen attendance behavior of *Nosema*-infected worker honey bees. J Econ Entomol 63: 1539–1541.

[26] Fries I (1989) Observations on the development and transmission of *Nosema apis* Z. in the ventriculus of the honeybee. J Apicult Res 28: 107–117.

[27] Fries I, Granados RR, Morse RA (1992) Intracellular germination of spores of *Nosema apis* Z. Apidologie 23: 61–70.

[28] Higes M, Martin R, Meana A (2006) *Nosema ceranae*, a new microsporidian parasite in honeybees in Europe. J Invertebr Pathol 92: 93–95. 16574143 10.1016/j.jip.2006.02.005

[29] Huang WF, Jiang JH, Chen YW, Wang CH (2007) A *Nosema ceranae* isolate from the honeybee *Apis mellifera* . Apidologie 38: 30–37.

[30] Klee J, Besana AM, Genersch E, Gisder S, Nanetti A, et al (2007) Widespread dispersal of the microsporidian *Nosema ceranae*, an emergent pathogen of the western honey bee, *Apis mellifera* . J Invertebr Pathol 96: 1–10. 17428493 10.1016/j.jip.2007.02.014

[31] Paxton RJ, Klee J, Korpela S, Fries I (2007) *Nosema ceranae* has infected *Apis mellifera* in Europe since at least 1998 and may be more virulent than *Nosema apis* . Apidologie 38: 558–565.

[32] Teixeira EW, dos Santos LG, Sattler A, Message D, Alves MLTMF, et al (2013) *Nosema ceranae* has been present in Brazil for more than three decades infecting Africanized honey bees. J Invertebr Pathol 114: 250–254. 10.1016/j.jip.2013.09.002 24025844

[33] Ferroglio E, Zanet S, Peraldo N, Tachis E, Trisciuoglio A, et al (2013) *Nosema ceranae* has been infecting honey bees *Apis mellifera* in Italy since at least 1993. J Apicult Res 52: 60–61. 24860952

[34] Williams GR, Shafer ABA, Rogers REL, Shutler D, Stewart DT (2008) First detection of *Nosema ceranae*, a microsporidian parasite of European honey bees (*Apis mellifera*), in Canada and central USA. J Invertebr Pathol 97: 189–192. 17897670 10.1016/j.jip.2007.08.005

[35] Forsgren E, Fries I (2010) Comparative virulence of *Nosema ceranae* and *Nosema apis* in individual European honey bees. Vet Parasitol 170: 212–217. 10.1016/j.vetpar.2010.02.010 20299152

[36] Higes M, Garcia-Palencia P, Martin-Hernandez R, Meana A (2007) Experimental infection of *Apis mellifera* honeybees with *Nosema ceranae* (Microsporidia). J Invertebr Pathol 94: 211–217. 17217954 10.1016/j.jip.2006.11.001

[37] Higes M, Martín-Hernández R, Botías C, Garrido Bailón E, González-Porto AV, et al (2008) How natural infection by *Nosema ceranae* causes honeybee colony collapse. Environ Microbiol 10: 2659–2669. 10.1111/j.1462-2920.2008.01687.x 18647336

[38] Higes M, Martin-Hernandez R, Garrido-Bailon E, Gonzalez-Porto AV, Garcia-Palencia P, et al (2009) Honeybee colony collapse due to *Nosema ceranae* in professional apiaries. Environ Microbiol Rep 1: 110–113. 10.1111/j.1758-2229.2009.00014.x 23765741

[39] Martin-Hernandez R, Meana A, Prieto L, Salvador AM, Garrido-Bailon E, et al (2007) Outcome of colonization of *Apis mellifera* by *Nosema ceranae* . Appl Environ Microbiol 73: 6331–6338. 17675417 10.1128/AEM.00270-07 PMC2075036

[40] Stevanovic J, Stanimirovic Z, Genersch E, Kovacevic SR, Ljubenkovic J, et al (2011) Dominance of *Nosema ceranae* in honey bees in the Balkan countries in the absence of symptoms of colony collapse disorder. Apidologie 42: 49–58.

[41] Stevanovic J, Simeunovic P, Gajic B, Lakic N, Radovic D, et al (2013) Characteristics of *Nosema ceranae* infection in Serbian honey bee colonies. Apidologie 44: 522–536.

[42] Gisder S, Hedtke K, Möckel N, Frielitz MC, Linde A, et al (2010) Five-year cohort study of *Nosema* spp. in Germany: Does climate shape virulence and assertiveness of *Nosema ceranae*? Appl Environ Microbiol 76: 3032–3038. 10.1128/AEM.03097-09 20228103 PMC2863439

[43] Fernández JM, Puerta F, Cousinou M, Dios-Palomares R, Campano F, et al (2012) Asymptomatic presence of *Nosema* spp. in Spanish commercial apiaries. J Invertebr Pathol 111: 106–110. 10.1016/j.jip.2012.06.008 22820066

[44] Williams GR, Shutler D, Burgher-MacLellan KL, Rogers REL (2014) Infra-population and -community dynamics of the parasites *Nosema apis* and *Nosema ceranae*, and consequences for honey bee (*Apis mellifera*) hosts. PLoS ONE 9: e99465 10.1371/journal.pone.0099465 24987989 PMC4079283

[45] Botías C, Martín-Hernández R, Barrios L, Meana A, Higes M (2013) *Nosema* spp. infection and its negative effects on honey bees (*Apis mellifera iberiensis*) at the colony level. Vet Res 44: 25 10.1186/1297-9716-44-25 23574888 PMC3640932

[46] Fenoy S, Rueda C, Higes M, Martín-Hernandez R, del Aguila C (2009) High-level resistance of *Nosema ceranae*, a parasite of the honeybee, to temperature and desiccation. Appl Environ Microbiol 75: 6886–6889. 10.1128/AEM.01025-09 19734329 PMC2772422

[47] Martin-Hernandez R, Meana A, Garcia-Palencia P, Marin P, Botias C, et al (2009) Effect of temperature on the biotic potential of honeybee microsporidia. Appl Environ Microbiol 75: 2554–2557. 10.1128/AEM.02908-08 19233948 PMC2675226

[48] Williams GR, Sampson MA, Shutler D, Rogers REL (2008) Does fumagillin control the recently detected invasive parasite *Nosema ceranae* in western honey bees (*Apis mellifera*)? J Invertebr Pathol 99: 342–344. 10.1016/j.jip.2008.04.005 18550078

[49] Williams GR, Shutler D, Little CM, Burgher-MacLellan KL, Rogers REL (2011) The microsporidian *Nosema ceranae*, the antibiotic Fumagilin-B (R), and western honey bee (*Apis mellifera*) colony strength. Apidologie 42: 15–22.

[50] Bailey L (1953) Effect of Fumagillin upon *Nosema apis* (Zander). Nature 171: 212 13036831 10.1038/171212a0

[51] Katznelson H, Jamieson CA (1952) Control of nosema disease of honeybees with fumagillin. Science 115: 70–71. 14913168 10.1126/science.115.2977.70

[52] Pajuelo AG, Torres C, Bermejo FJO (2008) Colony losses: a double blind trial on the influence of supplementary protein nutrition and preventative treatment with fumagillin against *Nosema ceranae* . J Apicult Res 47: 84–86.

[53] Huang WF, Solter LF, Yau PM, Imai BS (2013) *Nosema ceranae* escapes fumagillin control in honey bees. PLoS Pathog 9:e1003185 10.1371/journal.ppat.1003185 23505365 PMC3591333

[54] Botías C, Martín-Hernández R, Meana A, Higes M (2013) Screening alternative therapies to control Nosemosis type C in honey bee (*Apis mellifera iberiensis*) colonies. Res Vet Sci 95: 1041–1045. 10.1016/j.rvsc.2013.09.012 24148868

[55] Porrini MP, Audisio MC, Sabaté DC, Ibarguren C, Medici SK, et al (2010) Effect of bacterial metabolites on microsporidian *Nosema ceranae* and on its host *Apis mellifera* . Parasitol Res 107: 381–388. 10.1007/s00436-010-1875-1 20467753

[56] Maistrello L, Lodesani M, Costa C, Leonardi F, Marani G, et al (2008) Screening of natural compounds for the control of nosema disease in honeybees (*Apis mellifera*). Apidologie 39: 436–445.

[57] Underwood RM, Currie RW (2004) Indoor winter fumigation of *Apis mellifera* (Hymenoptera: Apidae) colonies infested with *Varroa destructor* (Acari: Varroidae) with formic acid is a potential control alternative in northern climates. J Econ Entomol 97: 177–186. 15154434 10.1093/jee/97.2.177

[58] Yoder JA, Jajack AJ, Cornacchione WS, Dunn AL, Cunningham EG, et al (2014) *In vitro* evaluation of sugar syrups, antibiotics, and miticides on growth of honey bee pathogen, *Ascosphaera apis*: Emphasis for chalkbrood prevention is on keeping bees healthy. Apidologie 45: 568–578.

[59] Genersch E, von der Ohe W, Kaatz H, Schroeder A, Otten C, et al (2010) The German bee monitoring project: a long term study to understand periodically high winter losses of honey bee colonies. Apidologie 41: 332–352.

[60] Olsen PE, Rice WA, Liu TP (1986) *In vitro* germination of *Nosema apis* spores under conditions favorable for the generation and maintenance of sporoplasms. J Invertebr Pathol 47: 65–73.

[61] Kobets T, Badalova J, Grekov I, Havelkova H, Svobodova M, et al (2010) Leishmania parasite detection and quantification using PCR-ELISA. Nat Prot 5: 1074–1080. 10.1038/nprot.2010.68 20539283

[62] Cornman RS, Chen YP, Schatz MC, Street C, Zhao Y, et al (2009) Genomic analyses of the microsporidian *Nosema ceranae*, an emergent pathogen of honey bees. PLoS Pathog 5 (6): e1000466 10.1371/journal.ppat.1000466 19503607 PMC2685015

[63] Katiyar SK, Visvesvara GS, Edlind TD (1995) Comparisons of ribosomal RNA sequences from amitochondrial protozoa: implications for processing, mRNA binding and paromomycin susceptibility. Gene 152: 27–33. 7828924 10.1016/0378-1119(94)00677-k

[64] Moffett JO, Lackett JJ, Hitchcock JD (1969) Compounds tested for control of *Nosema* in honey bees. J Econ Entomol 62: 886–889.

[65] Ding WQ, Liu B, Vaught JL, Palmiter RD, Lind SE (2006) Clioquinol and docosahexaenoic acid act synergistically to kill tumor cells. Mol Cancer Ther 5: 1864–1872. 16891473 10.1158/1535-7163.MCT-06-0067

[66] Ding WQ, Liu B, Vaught JL, Yamauchi H, Lind SE (2005) Anticancer activity of the antibiotic clioquinol. Cancer Res 65: 3389–3395. 15833873 10.1158/0008-5472.CAN-04-3577

[67] Beauvais B, Sarfati C, Challier S, Derouin F (1994) *In vitro* model to assess effect of antimicrobial agents on *Encephalitozoon cuniculi* . Antimicrob Agents Chemother 38: 2440–2448. 7840584 10.1128/aac.38.10.2440 PMC284758

[68] Katiyar SK, Edlind TD (1997) *In vitro* susceptibilities of the AIDS-associated microsporidian *Encephalitozoon intestinalis* to albendazole, its sulfoxide metabolite, and 12 additional benzimidazole derivatives. Antimicrob Agents Chemother 41: 2729–2732. 9420047 10.1128/aac.41.12.2729 PMC164197

[69] Haque MA, Hollister WS, Willcox A, Canning EU (1993) The antimicrosporidial activity of albendazole. J Invertebr Pathol 62: 171–177. 8228321 10.1006/jipa.1993.1092

[70] Bhat SA, Nataraju B, Bashir I (2012) Efficacy of different benzimidazole derivatives on microsporidiosis of lamerin breed of the silkworm, *Bombyx mori* L. J Entomol Nematol 4: 12–14.

[71] He Q, Leitch GJ, Visvesvara GS, Wallace S (1996) Effects of nifedipine, metronidazole, and nitric oxide donors on spore germination and cell culture infection of the microsporidia *Encephalitozoon hellem* and *Encephalitozoon intestinalis* . Antimicrob Agents Ch 40: 179–185. 8787902 10.1128/aac.40.1.179 PMC163079

[72] Johny S, Nimmo AS, Fisher MA, Inks ES, Kirkpatrick RM, et al (2009) Testing intra-hemocelic injection of antimicrobials against *Encephalitozoon* sp. (Microsporidia) in an insect host. Parasitol Res 104: 419–424. 10.1007/s00436-008-1214-y 18850113

[73] Payne MA, Baynes RE, Sundlof SE, Craigmill A, Webb AI, et al (1999) Drugs prohibited from extralabel use in food animals. J Am Vet Med Ass 215: 28–32. 10490381

[74] Johny S, Whitman DW, Bridge Study Group (2008) Effect of four antimicrobials against an *Encephalitozoon* sp (Microsporidia) in a grasshopper host. Parasitol Int 57: 362–367. 10.1016/j.parint.2008.03.002 18495525

[75] Vollenbroich D, Pauli G, Ozel M, Vater J (1997) Antimycoplasma properties and application in cell culture of surfactin, a lipopeptide antibiotic from *Bacillus subtilis* . Appl Environ Microbiol 63: 44–49. 8979337 10.1128/aem.63.1.44-49.1997 PMC168300

[76] Kowall M, Vater J, Kluge B, Stein T, Franke P, et al (1998) Separation and characterization of surfactin isoforms produced by *Bacillus subtilis* OKB 105. J Colloid Interf Sci 204: 1–8. 10.1006/jcis.1998.5558 9665760

[77] Peypoux F, Bonmatin JM, Wallach J (1999) Recent trends in the biochemistry of surfactin. Appl Microbiol Biotechnol 51: 553–563. 10390813 10.1007/s002530051432

[78] Seydlová G, Svobodová J (2008) Review of surfactin chemical properties and the potential biomedical applications. Cent Eur J Med 3: 123–133.

[79] Kim SD, Cho JY, Park HJ, Lim CR, Lim JH, et al (2006) A comparison of the anti-inflammatory activity of surfactin A, B, C, and D from *Bacillus subtilis* . J Microbiol Biotechnol 16: 1656–1659.

[80] Grangemard I, Peypoux F, Wallach J, Das BC, Labbe HC, et al (1997) Lipopeptides with improved properties: structure by NMR, purification by HPLC and structure-activity relationships of new isoleucyl-rich surfactins. J Peptide Sci 3: 145–154. 9230480 10.1002/(sici)1099-1387(199703)3:2<145::aid-psc96>3.0.co;2-y

